# Direct-to-implant breast reconstruction in prepectoral versus subpectoral planes: a meta-analysis framework for comparing complication rates and patient-reported outcomes

**DOI:** 10.1097/SP9.0000000000000054

**Published:** 2025-08-07

**Authors:** Yousef Tanas, Grace Gasper, Julie Tanas, Sarya Swed, Aldona Spiegel

**Affiliations:** a Institute for Reconstructive Surgery, Houston Methodist Hospital, Weill Cornell Medicine, Houston, Texas, United States; b Texas A&M School of Engineering Medicine, Houston, Texas, United States; c Alexandria Medical School, Alexandria, Egypt; d Faculty of Medicine, University of Aleppo, Syria

**Keywords:** breast reconstruction, implant, prepectoral, subpectoral, direct-to-, implant

## Abstract

**Background:**

Direct-to-implant (DTI) breast reconstruction has become a widely accepted approach for postmastectomy breast reconstruction. Traditionally, implants were placed in the subpectoral (SP) plane to maximize soft tissue coverage; however, recent advances in surgical technique and implant materials, such as acellular dermal matrices (ADMs) and meshes, have led to a resurgence in the prepectoral (PP) approach. Recent studies have shown conflicting evidence regarding their respective complication profiles and patient-reported outcomes. Thus, comprehensive head-to-head meta-analysis is needed to evaluate the safety and effectiveness of PP versus SP in DTI breast reconstruction.

**Methods:**

Following PRISMA guidelines, this systematic review and meta-analysis will compare complication rates and patient-reported outcomes between PP and SP in DTI reconstruction. MEDLINE (PubMed), Scopus, Web of Science, Cochrane Central Register of Controlled Trials, Cochrane Database of Systematic Reviews, and Clinicaltrials.org will be searched to identify comparative studies. Eligible studies must report at least one primary outcome, such as capsular contracture or surgical complications. Secondary outcomes will include BREAST-Q scores, pain scores, and length of hospital stay. Data will be extracted independently by two reviewers, and methodological quality will be assessed using appropriate risk of bias tools (ROBINS-I for nonrandomized studies and Rob 2 for randomized controlled trials). Meta-analysis will be performed using Review Manager 5.4, applying random-effects models in cases of significant heterogeneity. Subgroup and sensitivity analyses will be conducted where applicable.

**Discussion:**

This study aims to synthesize the current evidence comparing PP and SP in DTI breast reconstruction to inform surgical decision-making and optimize patient outcomes. The results will provide surgeons and patients with a clearer understanding of the benefits and risks associated with each reconstructive plane.

## Introduction

Implant-based breast reconstruction (IBBR) remains the most commonly performed method of postmastectomy reconstruction worldwide, accounting for over 80% of breast reconstructions in the United States alone^[1-3]^. Direct-to-implant (DTI) reconstruction (where a permanent implant is placed immediately following mastectomy without the use of a tissue expander) has gained popularity due to shorter treatment duration, fewer surgeries, and improved patient satisfaction^[4-6]^.

**HIGHLIGHTS**

- First meta-analysis directly comparing prepectoral versus subpectoral DTI breast reconstruction.
- Outcomes include surgical complications and patient-reported satisfaction (BREAST-Q scores).
- Registered on PROSPERO and following PRISMA-P guidelines.
- Meta-analysis will include all RCTs and observational studies for comprehensive evidence.
- It will provide surgeons with evidence-based guidance for technique selection.

Historically, DTI reconstruction was performed in the subpectoral (SP) plane to ensure adequate implant coverage and reduce the risk of complications, such as implant exposure and capsular contracture. Nonetheless, this technique is associated with its own drawbacks, including animation deformity, postoperative pain, and functional limitations^[7-10]^. With the advent of acellular dermal matrices (ADMs), refined mastectomy techniques, and improved implant technologies, the prepectoral (PP) approach has resurged in popularity^[11,12]^.

While PP DTI reconstruction offers potential advantages, such as reduced animation deformity and improved aesthetic outcomes, concerns remain about higher rates of implant malposition and capsular contracture. Several recent studies have compared outcomes between PP and SP approaches, but findings remain inconsistent due to heterogeneity in study design, sample size, follow-up duration, and outcome measures^[13–22]^.

Given the growing interest in PP reconstruction and the ongoing debate regarding its safety and efficacy compared to the SP approach, a comprehensive head-to-head meta-analysis will help objectively determine the most suitable approach. To our knowledge, this will be the first meta-analysis comparing surgical complications and patient-reported outcomes of DTI breast reconstruction performed in the PP versus SP planes.

## Methodology

**Figure 1.**
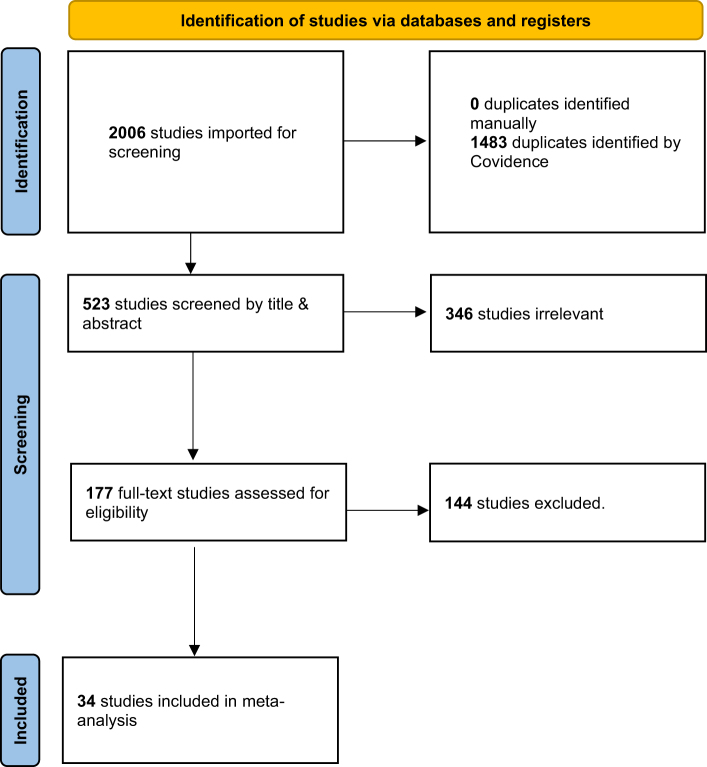
PRISMA flow diagram. Source: Page MJ, et al. BMJ 2021;372:n71. doi: 10.1136/bmj.n71. This work is licensed under CC BY 4.0. To view a copy of this license, visit https://creativecommons.org/licenses/by/4.0/

This review has been registered with the International Prospective Register of Systematic Reviews (PROSPERO), part of the National Institute for Health Research (NIHR) and the regulations of the preferred reporting items of systematic reviews and meta-analyses (PRISMA) will be followed^[23]^. This protocol followed the PRISMA-P guidelines for protocols^[24]^ (Supplementary Material 1 http://links.lww.com/ISJP/A14), the AMSTAR 2 guidelines^[25]^ (Supplementary Material 2 http://links.lww.com/ISJP/A15), and the TITAN Guideline Checklist 2025 (Supplementary Material 3 http://links.lww.com/ISJP/A16)^[26]^. A PRISMA Flow Chart has been generated as shown in Figure 1.

### Study question

In breast cancer survivors who have undergone DTI breast reconstruction, what are the efficacy and complication profiles of PP versus SP implant placement, and how do patient-reported outcomes compare between the two techniques?

### Literature search

A systematic literature search has been conducted with the following databases: using MEDLINE (PubMed), Scopus, Web of Science, Cochrane Central Register of Controlled Trials, Cochrane Database of Systematic Reviews, and Clinicaltrials.org. The search timeframe includes all studies from inception to search date. Search strategy keywords (conducted in English but with no restriction on including non-English papers) included the following terms in various combinations and forms:

Direct-to-implant, single-stage, or one-stage

Subpectoral, retropectoral, or subfascial

prepectoral

Breast reconstruction

Post-operative complications

BREAST-Q patient satisfaction

As with most systematic reviews, publication bias or potential for underreporting of negative results may be a limitation. Thus, other studies will be identified by contacting authors or experts, looking through all the articles that cite the papers included in the review (“snowballing”), reference list checking, searching conference proceedings, searching dissertation, and thesis databases and searching trial or study registers.

### Study selection and data extraction

Studies will be screened according to PICO criteria (Table 1), inclusion and exclusion criteria (Table 2) by two independent reviewers. Non-randomized (retrospective and prospective) studies and randomized control trials will be included. Titles and abstracts will be screened followed by full-text screening. Information from studies that pass initial and full text screening will be extracted by two independent reviewers and conflicts will be resolved by a third reviewer. Data will be extracted in into a spreadsheet. Outcome data will include capsular contracture, animation deformity, implant loss/failure, skin flap necrosis, wound dehiscence, delayed healing, explantation, infection, hematoma, seroma, drain time, patient satisfaction (BREAST-Q), drain time, and pain scores. Outcomes on oncologic safety (e.g. cancer recurrence) may also be assessed if reported in at least three included studies. Demographics data will include age, body mass index, diabetes, smoking status, radiotherapy, chemotherapy, and follow up time.

**Table 1:** PICO criteria for study selection

Population	Breast cancer survivors undergoing DTI breast reconstruction DTI must be either PP or SP
Intervention	DTI in the PP plane
Comparison	DTI in the SP plane
Outcome	Surgical complications capsular contracture, animation deformity, implant loss/failure, skin flap necrosis, wound dehiscence, delayed healing, explantation, infection, hematoma, seroma) Pain scores BREAST-Q Patient Satisfaction Drain time

**Table 2:** Inclusion and exclusion criteria for study selection

Inclusion criteria	Observational or interventional studies comparing SP and PP DTI breast reconstruction. Studies reporting complication rates, pain scores, oncologic safety, drain time, or BREAST-Q patient satisfaction outcomes for both arms
Exclusion criteria	Studies where both SP and PP planes were not compared Studies comparing the two planes but performed in two stages using a tissue expander instead of DTI Reviews, letters to the editor, commentaries, and editorials Studies with insufficient data on complication rates or patient satisfaction Studies where patients were switched from one plane to the other

### Study quality

Study quality and risk of bias will be assessed using the Cochrane ROB 2 score for randomized control trials and ROBINS-I tool for non-randomized studies. Studies will be independently reviewed by two authors and conflicts discussed with a third independent reviewer. Kappa statistics (with a threshold of ≥0.8) will be calculated to assess inter-rater reliability. Any amendments to the protocol will be documented via updates on PROSPERO and reflected in the final systematic review manuscript.

### Statistical analysis

Meta-analysis will be performed using Review Manager 5.4 software. Dichotomous data with outcomes comparing complication rates will be presented as risk ratios (RR) with their respective 95% confidence intervals (CI) using the Mantel–Haenszel method. Continuous data with outcomes on drain time and BREAST-Q patient satisfaction will be presented as mean differences (MD) with their respective CI using the inverse variance method. Heterogeneity will be assessed using *I*^2^ statistics. A random-effects model will be applied in case of significant heterogeneity followed by sensitivity analysis. Forest plots will be created for two arm studies, including heterogeneity and *P*-value for overall effect. A *P*-value less than 0.05 will be considered significant. Subgroup analyses on types of reconstruction (e.g. SP or PP), radiotherapy, chemotherapy, and different types of mastectomies will be performed if there will be enough data. Publication bias will be assessed using funnel plots and Egger’s test where an outcome has been reported by 10 or more studies^[27]^.

## Discussion

DTI breast reconstruction is increasingly preferred for its efficiency, reduced surgical burden, and aesthetic advantages. Nonetheless, the optimal plane of implant placement remains debatable. The PP technique avoids muscle dissection, reducing postoperative pain and animation deformity, but raises concerns regarding implant visibility and potential for complications such as capsular contracture^[20,28]^.

Recent studies have shown mixed findings. Abbas et al. observed a higher rate of secondary procedures in SP reconstructions, including implant exchange and pocket revision, though fat grafting needs were comparable across both approaches^[22]^. Similarly, Zhang et al. reported fewer complications (regarding pain and capsular contracture) in the PP group, alongside improved physical and mental health scores^[14]^.

In contrast, Fraser et al. found higher psychosocial well-being scores in patients undergoing subpectoral reconstruction, suggesting that the muscle coverage may provide a psychological benefit for certain patients despite increased physical discomfort^[18]^. Dyrberg et al., in a randomized controlled trial, found no statistically significant difference in patient-reported outcomes (BREAST-Q scores) between PP and SP placements, although both techniques improved postoperative sexual well-being^[19]^.

Radiation therapy further complicates this decision. Naoum et al. demonstrated that PP and SP implant placements had comparable complication and reconstruction failure rates even in the setting of postmastectomy radiation, supporting the viability of PP DTI in oncologically complex cases^[16]^. However, Bassi et al. highlighted a slightly higher reoperation rate in PP reconstructions performed without ADM, suggesting a learning curve or potential vulnerability when ADM is omitted^[21]^.

In terms of patient satisfaction, Cogliandro et al. found significantly better BREAST-Q scores (including psychosocial, sexual, and physical well-being) for PP reconstruction, with lower complication and reoperation rates^[20]^. Ibraheem et al. further reinforced the cost-effectiveness and safety of PP DTI using Ultrapro® mesh in low-resource settings, offering a pragmatic alternative to expensive ADMs without compromising outcomes^[28]^.Taken together, current literature supports the use of both approaches but with conflicting evidence regarding complication rates and patient-reported outcomes. Variables such as BMI, radiation status, use of ADM, and surgeon experience may also influence the success of either technique^[13]^. To our knowledge, this will be the first meta-analysis that aims to systematically evaluate the available evidence with the use of subgroup analyses to provide clearer objective guidance to surgeons in choosing the optimal plane for DTI breast reconstruction. Future research should also explore the application of AI in breast reconstruction, including the development of image-based and multimodal datasets that could predict complication risks and optimize surgical outcomes in breast cancer survivors^[29–31]^.

### Learning points

- This will be the first meta-analysis of all head-to-head comparative studies comparing SP vs PP implant placement when performed as DTI.
- Our meta-analysis will clarify whether one technique is better than the other or if either technique yields similar outcomes with regard to complication rates and patient satisfaction.

We will perform subgroup analyses where appropriate (particularly for ADMs) to eliminate potential confounding and determine if there are other factors that may contribute to better outcomes with either technique.

## Data Availability

Not applicable.

## Research registration unique identifying number (UIN)

Systematic review registration: PROSPERO CRD420251012220.
